# Long-term clinical outcomes for patients with uncrossable patent foramen ovale

**DOI:** 10.3389/fcvm.2023.1249259

**Published:** 2023-10-13

**Authors:** Yonghoon Shin, Albert Youngwoo Jang, Yoonsun Won, Taeil Yang, Joohan Kim, Joonpyo Lee, Jeongduk Seo, Minsu Kim, Pyung Chun Oh, Wook-Jin Chung, Jeonggeun Moon, Woong Chol Kang

**Affiliations:** ^1^ Department of Critical Care Medicine, Samsung Medical Center, Sungkyunkwan University School of Medicine, Seoul, Republic of Korea; ^2^ Division of Cardiology, Department of Internal Medicine, Gil Medical Center, Gachon University, Incheon, Republic of Korea; ^3^ Department of Internal Medicine, Cardiovascular Center, Chinjujeil Hospital, Jinju, Republic of Korea

**Keywords:** patent foramen ovale, uncrossable PFO, stroke, transient ischemic attack, percutaneous PFO closure

## Abstract

**Introduction:**

Patent foramen ovale (PFO) closure is performed in selected patients with cryptogenic stroke to prevent recurrence. The prognosis of patients with uncrossable PFO after failed guidewire or catheter passage during the procedure remains unknown. We compared the long-term prognosis between uncrossable PFO and successful PFO closure in patients with high-grade PFO shunts.

**Methods:**

We analyzed patients who underwent PFO closure for stroke or transient ischemic attack (TIA) prevention at Gachon University Gil Medical Center between April 2010 and March 2022. The primary outcome was a composite of recurrent stroke or TIA. Secondary outcomes included stroke, TIA, all-cause death, and a composite of stroke, TIA, and all-cause death.

**Results:**

Of 286 patients, 245 were included in the analysis after excluding those with transseptal puncture technique usage or concurrent atrial septal defect. Among them, 82 had uncrossable PFO, and 163 underwent successful PFO closure. Large shunts were more prevalent in the PFO closure group compared to the uncrossable PFO group (62.0% vs. 34.1%, *P* < 0.001), and resting shunts were also more common in the PFO closure group (17.8% vs. 2.4%, *P* < 0.001). Stroke or TIA occurred in 2 patients (2.4%) in the uncrossable PFO group and 8 patients (4.9%) in the PFO closure group (hazard ratio, 1.44; 95% confidence interval, 0.30–6.81; *P* = 0.647). Additionally, no disparities in the occurrence of stroke or TIA were found in subgroups divided by baseline characteristics, RoPE score, or shunt grade.

**Conclusion:**

Clinical outcomes for patients with uncrossable PFO seem similar to those with successful PFO closure.

## 1. Introduction

Paradoxical embolism due to patent foramen ovale (PFO)-mediated right to left shunting is considered a contributing factor to cryptogenic stroke (CS) (1–4). In patients with high-grade PFO shunts, the recurrence of ischemic stroke or transient ischemic attack (TIA) is significantly reduced in those who undergo PFO closure compared to those receiving medical-only treatment (5–8). Consequently, current guidelines recommend PFO closure for young patients with CS and high-grade PFO shunts (9).

**Figure 1:**
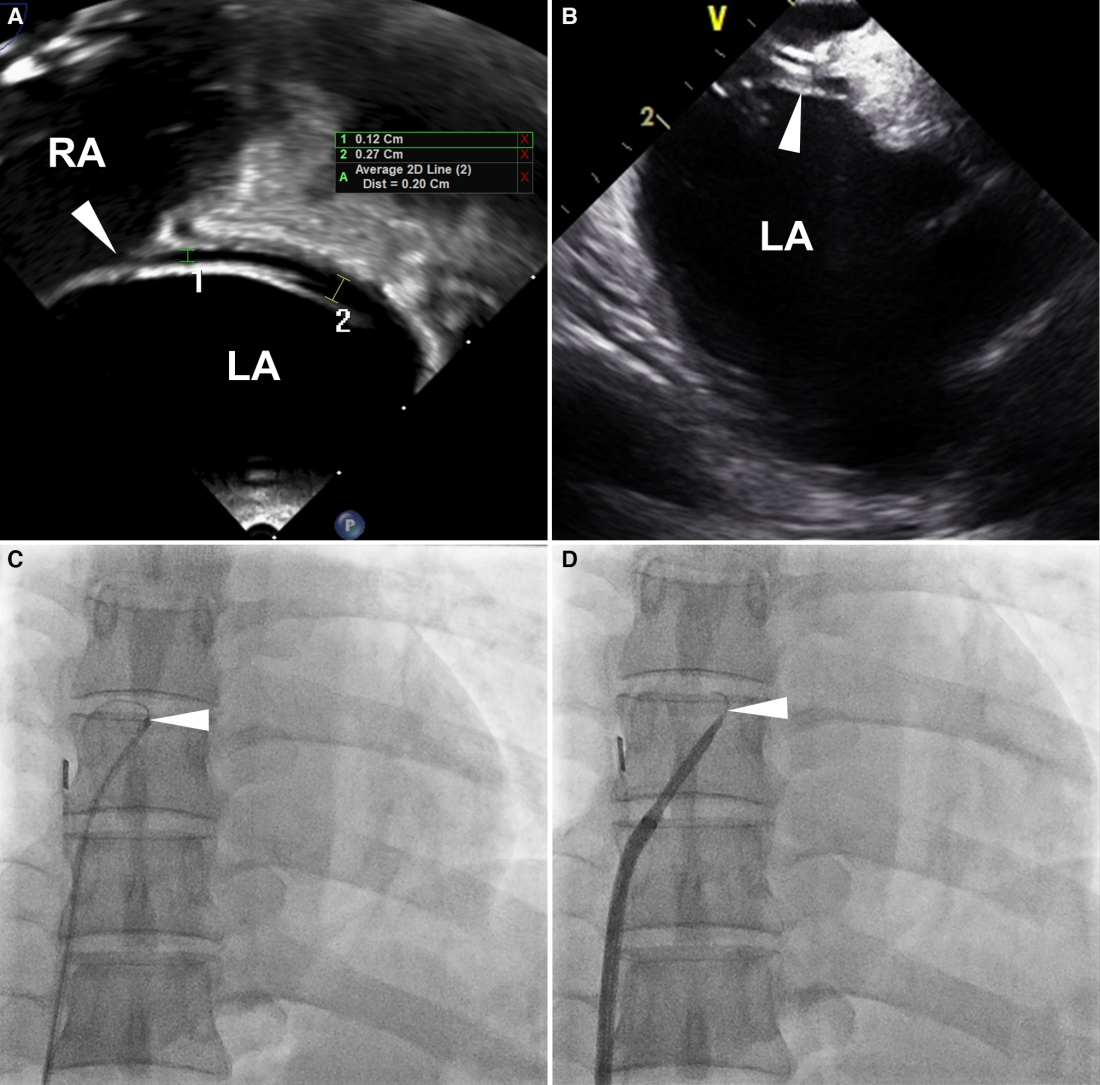
Definition of uncrossable PFO. Images from a 53-year-old man with an uncrossable PFO. (**A**) TEE 90-degree bicaval view shows a long-tunnel PFO. The probe was additionally rotated to optimize the visibility of the PFO tunnel in the bicaval view. (**B**) ICE imaging for PFO closure guidance. (**C**) 6 Fr MP catheter and 0.035-inch guidewire failed to pass through PFO. (**D**) SL1TM sheath replacement didn't resolve the issue. White arrowheads indicate PFO. ICE, intracardiac echocardiography; LA, left atrium; MP, multipurpose; PFO, patent foramen ovale; RA, right atrium; TEE, transesophageal echocardiography.

For PFO closure, a soft-tipped 0.035-inch guidewire or a multipurpose (MP) catheter is introduced through the PFO tunnel from the right atrium (RA) to the left atrium (LA) (10–13). However, in some cases, the guidewire or catheter fails to traverse the PFO tunnel, even in patients with high-grade shunts (Figure 1). In these patients, transseptal puncture around the PFO tunnel can be performed to facilitate wire or catheter passage (14–17). Nonetheless, our previous data indicate a higher incidence of recurrent stroke or TIA and residual shunt with this technique compared to the standard PFO closure technique (18). Consequently, transseptal puncture is an invasive procedure with a potential for poor outcomes, and its use in the treatment of uncrossable PFOs may be carefully considered.

Currently, there is a scarcity of data concerning the characteristics and long-term clinical outcomes of patients who experience unsuccessful PFO closure due to the inability to pass through the tunnel. In this study, we aimed to determine the long-term outcomes of patients with failed wire or catheter passage through the PFO tunnel, even when PFO closure was indicated.

## 2. Methods

### 2.1. Participants

We conducted a retrospective analysis of electronic medical records and echocardiographic data from the Gil Medical Center PFO registry in South Korea between April 2010 and March 2022. The inclusion criteria were as follows: (1) patients admitted to the neurology department with a diagnosis of stroke or TIA and referred to cardiology for evaluation of CS; (2) patients with PFO and a high-grade shunt, defined as grade 2 or 3, as confirmed on transesophageal echocardiography (TEE) with agitated saline test; and (3) patients in whom PFO closure was attempted. The decision to perform the procedure was determined by the heart-brain team (interventional cardiologist, cardiac imaging specialist, and neurologist) based on clinical data, echocardiographic findings, and patient preference. Exclusion criteria were as follows: (1) presence of concurrent atrial septal defect (ASD) and (2) transseptal puncture-assisted PFO closure. The current study conformed to the Declaration of Helsinki (sixth revision). The Institutional Review Board of Gil Medical Center, Gachon University College of Medicine approved this study (GDIRB 2014-35, 24 February 2014), and all patients provided written informed consent before enrollment.

### 2.2. Echocardiography

The TEE test with and without the Valsalva maneuver, along with the agitated saline test, was performed to confirm the presence and morphological characteristics of the PFO, including atrial septal aneurysm, shunt at rest, and the degree of the shunt. Atrial septal aneurysm was defined as a bulging of at least 10 mm beyond the atrial septum into either the RA or LA (8). PFO-induced right to left shunt and shunt at rest refer to agitated saline contrast appearing in the LA within three cardiac cycles in patients with RA opacification during the Valsalva maneuver and normal respiration, respectively (18–20). In addition to the criteria outlined above, a resting shunt was also defined by the presence of color Doppler flow crossing the PFO tunnel from right to left, visually observed on TEE even before the administration of agitated saline contrast. Shunting was defined as grade 1 if 3–9 contrast bubbles appeared, grade 2 if 10–30 contrast bubbles appeared, and grade 3 if more than 30 contrast bubbles appeared in the LA (16). Shunts classified as grade 2 or 3 are defined as high-grade, and closure seems to be effective in these patients (8, 9).

### 2.3. PFO closure procedure and definition of uncrossable PFO

PFO closure was performed under general or local anesthesia. After femoral venous access was achieved, anticoagulation was initiated with 5,000 units of intravenous unfractionated heparin. Additional heparin was administered throughout the procedure to maintain an activated clotting time ≥250s. We used a 6 Fr MP catheter (INFINITI THRULUMEN®, Cordis, Miami Lakes, FL, USA) and a 0.035-inch angled Terumo hydrophilic wire (Radifocus® Guidewire M, Terumo Medical, Tokyo, Japan) to pass through the PFO tunnel. The guidewire was advanced with repeated back-and-forth movements through the MP catheter. When unsuccessful, the guiding catheter was substituted with an 8 Fr standard Mullins™ (Medtronic, Minneapolis, MN, USA) or Swartz™ SL1 sheath (Abbott, Chicago, IL, USA) for better support. If guidewire or catheter passage still failed despite the substitution, we defined it as an uncrossable PFO and terminated the procedure (Figure 1). After successful passage to the LA, the guidewire and catheter were advanced to the left superior pulmonary vein. The guidewire was then substituted with a 0.035-inch Amplatz extra-stiff wire (Cook Medical, Bloomington, IN, USA). An 8 or 9 Fr guiding sheath was inserted into the PFO, and the closure device was deployed. After the procedure, the suggested antiplatelet treatment consisted of taking 100 mg of aspirin and 75 mg of clopidogrel daily for a minimum of three months. Between 1 and 3 months post-procedure, either follow-up transthoracic echocardiography or a TEE with an agitated saline test was conducted.

### 2.4. Definition of study endpoints

The primary endpoint was the occurrence of recurrent ischemic events, specifically ischemic stroke or TIA after the procedure. Hemorrhagic strokes were not included in this endpoint. Secondary endpoints included stroke, TIA, all-cause death, and a composite of stroke, TIA, and all-cause death.

### 2.5. Statistical analysis

Data are presented as means and standard deviations and were compared using independent sample *t*-tests for continuous variables. Chi-square tests were employed to compare categorical variables. Survival curves were estimated using the Kaplan-Meier method and compared with the log-rank test. Cox proportional hazard models were utilized to calculate hazard ratios, 95% confidence intervals (CI), and corresponding *P*-values. A stepwise multivariable Cox analysis was performed. Covariates that were significant in the univariate analysis or those with clinical relevance were included in the calculation of the multivariable-adjusted hazard ratio (HR_adj_). Multivariate linear regression analysis was conducted to exclude variables with multicollinearity. Statistical significance was set at *P* < 0.05. Statistical analyses were carried out using SPSS (version 22; SPSS Inc., Chicago, IL, USA).

## 3. Results

### 3.1. Patient inclusion and PFO closure success rate

**Figure 2:**
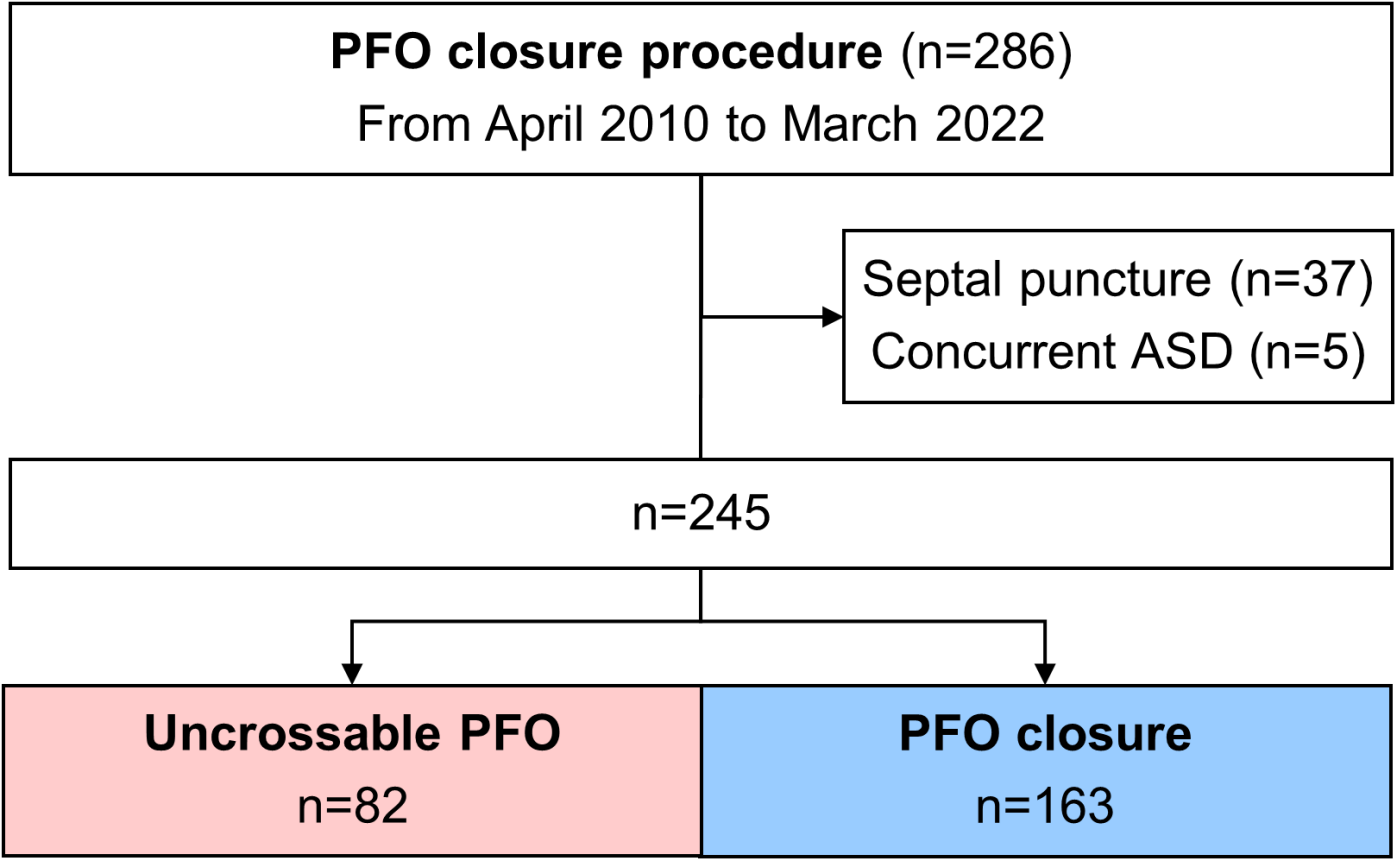
Flow diagram of the separation into groups. ASD, atrial septal defect; PFO, patent foramen ovale.

Between April 2010 and March 2022, 286 consecutive patients with CS, in whom PFO closure was attempted, were enrolled. A total of 245 patients were analyzed after excluding 41 patients (37 patients in whom PFO device was implanted using the transseptal puncture technique and 5 patients who had concurrent ASD). Of the 245 patients, 163 (66.5%) underwent successful PFO closure and 82 (33.5%) had uncrossable PFOs (Figure 2). The Amplatzer PFO Occluder (St Jude Medical, St. Paul, MN), GORE® Septal Occluder (WL Gore & Associates, Inc., Newark, DE), and Occlutech Figulla® Flex II (Jena, Germany) were implanted in all patients in the PFO closure group. All unsuccessful procedures were due to uncrossable PFOs.

### 3.3. Demographic data

The demographic data of the patients are shown in Table 1. Baseline characteristics were nearly similar between the two groups. However, a history of previous TIA and deep vein thrombosis were more frequent in the uncrossable PFO group than in the PFO closure group (Table 1). A large shunt (PFO grade 3) was more common in the PFO closure group than in the uncrossable PFO group (34.1% vs. 62.0%, *P* < 0.001). There were significantly more shunts at rest in the PFO closure group than in the uncrossable PFO group (2.4% vs. 17.8%, *P* < 0.001) (Table 1). There was no difference in the presence of atrial septal aneurysm between the two groups. The median follow-up days post-procedure were 719 days in the uncrossable PFO group and 926 days in the PFO closure group (*P* = 0.008). The PFO closure success rate was 53.4% (62/116) and 78.3% (101/129) for PFO grades 2 and 3, respectively (Supplementary Table S1).

**Table 1:** Baseline patient characteristics.

	Uncrossable PFO (*N* = 82)	PFO Closure (*N* = 163)	*P*
Demographic data			
Age, year	51.8 ± 10.8	51.4 ± 11.2	0.808
Male sex, no. (%)	63 (76.8)	125 (76.7)	0.980
Non-smoker. no./total no. (%)	7/16 (43.8)	50/104 (48.1)	0.747
Previous medical history, no. (%)			
Stroke	74 (90.2)	157 (96.3)	0.053
TIA	24 (29.3)	27 (16.6)	0.021
Recurrent stroke or TIA^a^	12 (14.6)	26 (16.0)	0.788
Hypertension	50 (61.0)	104 (63.8)	0.666
DM	18 (22.0)	34 (20.9)	0.844
Dyslipidemia	33 (40.2)	80 (49.1)	0.190
Coronary artery disease	2 (2.4)	9 (5.5)	0.272
Heart failure	6 (7.3)	19 (11.7)	0.290
COPD	3 (3.7)	5 (3.1)	0.806
DVT	5 (6.1)	2 (1.2)	0.031
PTE	0 (0.0)	2 (1.2)	0.314
Nature of stroke			
RoPE score	3.91 ± 1.53	4.18 ± 1.71	0.229
RoPE ≥6 — no. (%)	11 (13.4)	35 (21.5)	0.128
Echocardiographic Findings			
PFO Grade			<0.001
2	54 (65.9)	62 (38.0)	
3	28 (34.1)	101 (62.0)	
Resting shunt	2 (2.4)	29 (17.8)	0.001
Atrial septal aneurysm	1 (1.2)	3 (1.8)	0.717
Intracardiac thrombus	1 (1.2)	0 (0.0)	0.158
Post procedure follow-up			
Follow-up, days	719 (274–1,377)	926 (495–2,295)	0.008
Aspirin	73 (89.0)	94 (93.1)	0.484
Clopidogrel	60 (73.2)	130 (80.2)	0.274

Data presented as mean ± standard deviation (*N*), median (IQR), or *n*/*N* (%).

^a^ A Recurrent stroke or TIA refers to two or more stroke or TIA before the index procedure.

TIA, transient ischemic attack; DM, diabetes mellitus; COPD, chronic obstructive pulmonary disease; DVT, deep vein thrombosis; PTE, pulmonary thromboembolism; RoPE score, risk of paradoxical embolism score, PFO, patent foramen ovale.

### 3.3. Clinical outcomes

**Figure 3:**
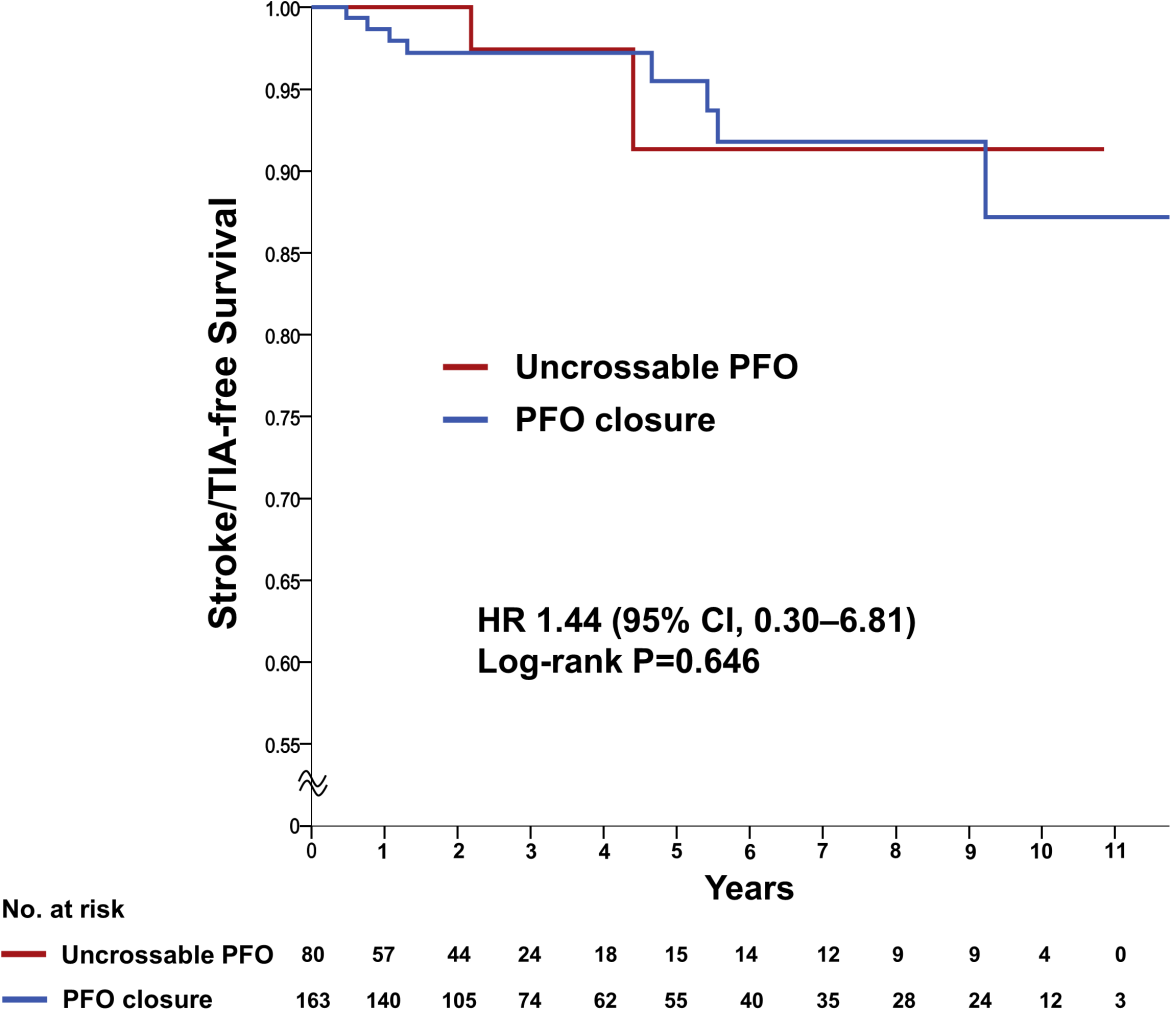
Primary outcome of recurrent ischemic stroke/TIA. Kaplan-Meier estimating the cumulative incidence of recurrent ischemic stroke or TIA in a time-to-first-event analysis. CI, confidence intervals; HR, hazard ratio; PFO, patent foramen ovale; TIA, transient ischemic attack.

The rate of recurrent stroke/TIA was similar between the groups (two patients [2.4%] in the uncrossable PFO group and eight patients [4.9%] in the PFO closure group) in the crude analysis (HR 1.44, 95% CI 0.30–6.81, *P* = 0.647) and after multivariable adjustment (HR_adj_ 1.27, 95% CI 0.25–6.42, *P* = 0.773) (Table 2 and Figure 3). The multivariable Cox analysis was adjusted for the presence of hypertension, diabetes mellitus, age ≥65 years, previous recurrent stroke or TIA, risk of paradoxical embolism (RoPE) score ≥6, and PFO grade. There was no difference in the occurrence of individual secondary endpoints, such as stroke, TIA, all-cause death, and composite of stroke/TIA and all-cause death, with or without adjustment (Table 2). Multivariate Cox regression analysis, using baseline characteristics, features of PFO, and procedural characteristics, revealed that recurrent stroke/TIA, namely in patients who had two or more strokes or TIAs prior to PFO closure, appeared as independent prognostic factors for stroke/TIA recurrence after PFO closure. The degree of PFO shunt and the uncrossable PFO were not significant factors (HR_adj_ 6.29, 95% CI 1.56–25.13, *P* = 0.009, model 1) (Table 3).

**Table 2:** Unadjusted and adjusted cox proportional hazard model according to procedure success.

		Uncrossable PFO, *n* (%)	PFO Closure, *n* (%)	HR	95% CI	*P*
Stroke/TIA	Unadjusted	2 (2.4)	8 (4.9)	1.44	0.30–6.81	0.647
	Adjusted^a^	–	–	1.27	0.25–6.42	0.773
Stroke	Unadjusted	0 (0)	6 (3.7)	34.15	0.02–INF	0.360
	Adjusted	–	–	INF	0.00–INF	0.973
TIA	Unadjusted	2 (2.4)	2 (1.2)	0.33	0.05–2.34	0.265
	Adjusted	–	–	0.22	0.02–1.98	0.175
Death	Unadjusted	1 (1.2)	2 (1.2)	0.64	0.06–7.08	0.714
	Adjusted	–	–	0.07	0.00–2.16	0.129
Stroke/TIA/Death	Unadjusted	3 (3.7)	9 (5.5)	1.06	0.29–3.93	0.933
	Adjusted	–	–	0.79	0.20–3.14	0.734

TIA, transient ischemic attack; INF, infinite; PFO, patent foramen ovale; HR, hazard ratio; CI, confidence interval.

^a^ Adjusted for hypertension, diabetes mellitus, age >65 years, previous recurrent stroke or TIA, RoPE score ≥6, and PFO grade.

**Table 3:** Multivariate cox regression analysis of recurrent ischemic stroke or transient ischemic attack.

	Model 1			Model 2		
	HR_adj_	95% CI	*P*	HR_adj_	95% CI	*P*
Uncrossable PFO	0.79	0.25–6.42	0.773	0.91	0.21–5.79	0.914
Age ≥65 year	2.15	0.24–19.11	0.492	1.93	0.21–17.94	0.562
Hypertension	1.79	0.25–13.04	0.567	1.86	0.27–12.67	0.528
DM	2.57	0.60–10.99	0.203	2.29	0.53–9.99	0.269
Previous recurrent stroke/TIA^a^	6.29	1.56–25.13	0.009	6.37	1.59–25.56	0.009
RoPE score ≥6	2.05	0.26–15.94	0.492	1.89	0.25–14.11	0.534
PFO grade	1.50	0.40–5.70	0.549			
Shunt at rest				2.82	0.65–12.28	0.168

PFO, patent foramen ovale; DM, diabetes mellitus; RoPE score, risk of paradoxical embolism score; HR_adj_, multivariable-adjusted hazard ratio; CI, confidence interval.

^a^ Previous recurrent stroke/TIA refers to two or more strokes or TIA before the index procedure.

### 3.4. Subgroup analysis

**Figure 4:**
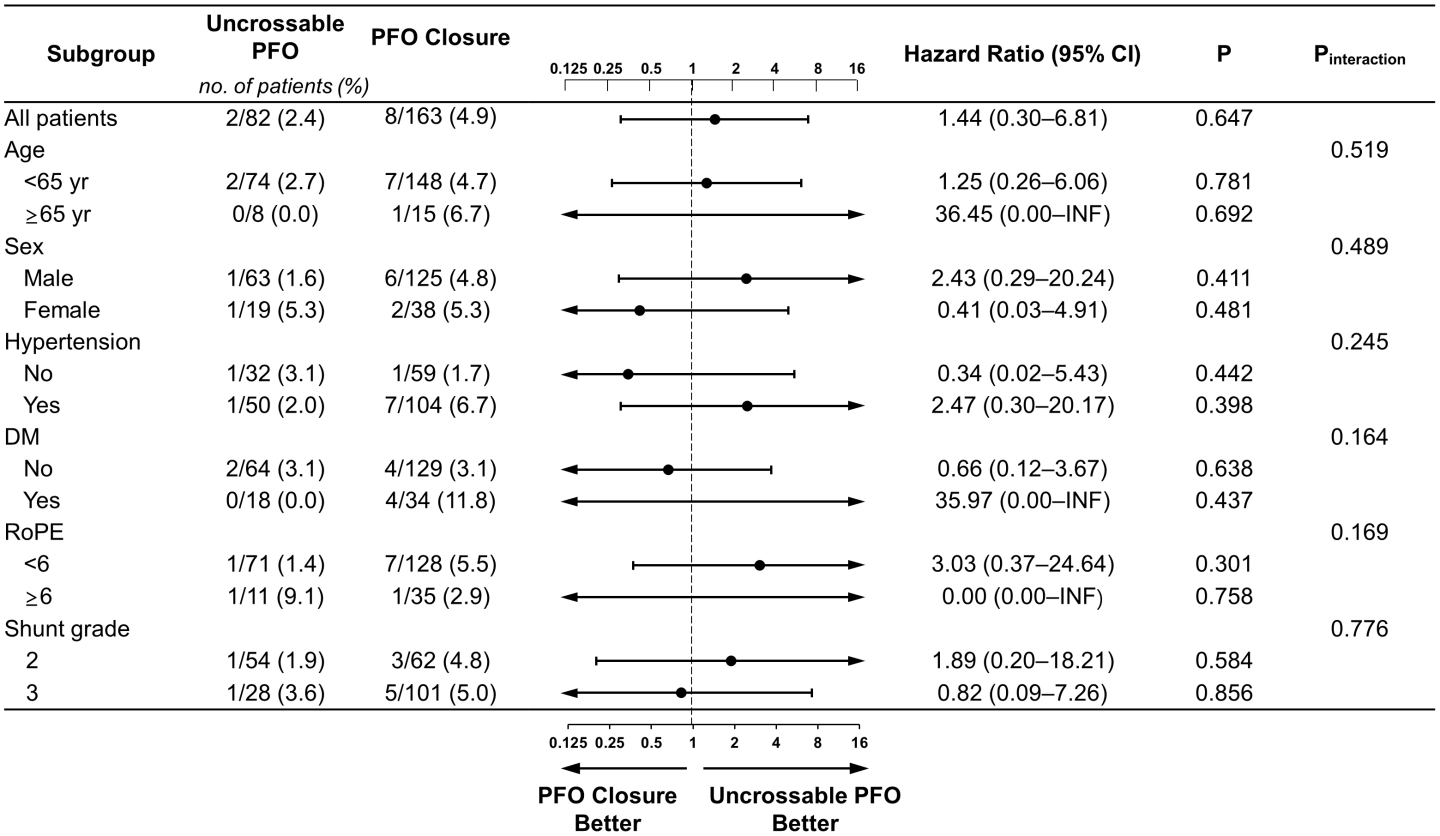
Subgroup analyses of primary outcome. Forest plot displaying hazard ratios for primary outcome events across prespecified subgroups. The dashed vertical line signifies the null hypothesis of no procedural effects. DM, diabetes mellitus; INF, infinite; TIA, transient ischemic attack.

There were no differences in recurrent stroke/TIA between the two groups regardless of age, sex, hypertension, diabetes, or the RoPE score. Regardless of the cutoff for PFO grade 2 or 3, there was no difference in the outcomes between the uncrossable PFO and PFO closure groups (Figure 4 and Supplementary Figure S1). There was no interaction between the subgroups and PFO closure success rate.

## 4. Discussion

This study addresses the fundamental question of whether it is safe to leave an uncrossable PFO untreated in patients with high-grade PFO shunts. To our knowledge, this is the first study to compare the prognosis of uncrossable PFO to the PFO closure. We found that: (1) the long-term clinical outcomes of the uncrossable PFO group were similar to those of the PFO closure group, (2) these outcomes were consistent regardless of the baseline PFO grade, and (3) recurrent stroke/TIA before the procedure is a strong prognostic factor for the stroke/TIA after procedure.

The proportion of uncrossable PFO population may have been overlooked. The incidence of uncrossable patent foramen ovale (PFO) varies considerably, ranging from 10%–40% in various studies (15, 21–23). According to existing literature on echocardiographic analysis in patients undergoing PFO closure, 54.5% exhibited complex PFOs, encompassing characteristics such as long-tunnel PFOs, a substantial Eustachian ridge, or an overly redundant Chiari network, which could potentially result in uncrossable PFO (24). Given that the transseptal puncture technique is more commonly used in these complex PFOs, it is reasonable to anticipate an occurrence of uncrossable PFOs within this category. Some studies that reported higher procedure success rates utilized the transseptal puncture technique, a method we intentionally did not employ in our study. Many papers neither discuss how they addressed uncrossable PFO nor report its incidence. Most PFO-related studies only compare PFO closure groups with medication-only groups, potentially neglecting uncrossable PFO. Thus, the true incidence of uncrossable PFOs in real-world clinical practice may be higher than our estimates, particularly if transseptal puncture techniques are not utilized and the incidence of complex PFOs is taken into account. The identified incidence of uncrossable PFO in our study is 33%, and although its exact alignment with actual incidence remains unclear, this figure falls within the range reported in existing literature. It should be emphasized that candidates for PFO closure were carefully selected after consultation with neurologists and cardiac imaging specialists, and all procedures were performed by highly experienced operators. Given our meticulous selection of PFO closure candidates and the skilled execution of procedures, we believe this to be acceptable. Additionally, demographic differences, such as the proportion of grade 3 PFOs, between our study and those reporting higher procedure success rates must also be considered.

Our study suggests that among patients undergoing a PFO closure procedure with high-grade PFO shunts, the prognostic implications of grade 2 vs. grade 3 shunts are not significant. Neither the difference between PFO grades 2 and 3 nor the presence or absence of a resting shunt emerged as independent predictors in a multivariate Cox regression analysis for recurrent stroke or TIA after the procedure. Furthermore, a subgroup analysis comparing the uncrossable PFO group to the PFO closure group, while considering PFO shunt grades 2 and 3, showed no difference in the primary endpoint. High-grade PFO shunts are reportedly associated with a higher risk of stroke recurrence, and PFO closure reduces this risk (8, 25–30). The leading randomized controlled trials, the Gore REDUCE and the RESPECT (Randomized Evaluation of Recurrent Stroke Comparing PFO Closure to Established Current Standard of Care Treatment) trials, did not demonstrate a significant difference in prognosis between the PFO closure group and the medication-only group when including PFO shunt grade 1 (5, 7). No existing studies have focused solely on comparing PFO shunt grades 2 and 3 while excluding grade 1. For this reason, our study chose to exclude patients with PFO shunt grade 1 as candidates for the procedure and included only high-grade PFO shunts.

In our study, several PFO tunnels were not crossable with a wire or catheter, even at baseline PFO grade 3 (28/82, 34.1%). These findings contradict the assumption that a larger shunt, defined as a large number of agitated saline microbubbles observed within three cycles, may indicate a larger PFO defect. The agitated saline test indirectly estimates the PFO size based on the number of microbubbles. It does not directly measure the PFO size (16). Several studies have reported that the PFO shunt grade reportedly does not correlate with the actual size PFO size (29, 31, 32). Thus, the estimated PFO shunt size may not be accurate, which may explain the uncrossable tunnels encountered in patients with grade 3 PFO. Anatomical complexities of the PFO, such as the long tunnel or the angle between the guiding catheter and PFO tunnel, have been previously elucidated (8, 14, 17, 22, 23, 33). We believe that regardless of the initial shunt severity, stroke incidence may be comparable to the crossable group wherein patients are inserted with the closure device. This might be because anatomical barriers, such as long-tunnel PFOs, a prominent Eustachian ridge, or a redundant Chiari network (24), may prevent emboli, thrombi, or linear structures like guidewires from passing through the PFO while still allowing smaller microbubbles to traverse. The relationship between these anatomical complexities and passage of wire or catheter requires further investigation.

We found that a previous history of recurrent stroke/TIA was the only indicator for future episodes of recurrent stroke/TIA. Patients with a previous history of recurrent stroke/TIA continued to be at a high risk for recurrent stroke/TIA even after successful PFO closure (HR_adj_ 11.08, 95% CI 2.05–59.98, *P* = 0.005). This indicates that the cause of stroke/TIA in patients with PFO who underwent closure has not been thoroughly evaluated. Although we tried to rule out other causes of stroke before the procedure, there was a possibility of missing causal factors such as atrial fibrillation (AF). In our study, three new-onset AF cases were noted after successful device implantation; one patient had persistent AF and two had paroxysmal AF. It is difficult to accurately determine whether AF in these patients was a device-induced complication or had gone undetected before the procedure. Regardless of the cause, AF is a strong risk factor for stroke or TIA.

There are some important limitations to this study. One of the most significant is the relatively high uncrossable rate of 33%. While this rate is higher compared to established trials such as Gore-REDUCE, it is crucial to take into account the patient exclusion criteria in those trials (7). Unlike these trials, our study did not exclude patients with anatomically complex PFO that have a higher likelihood of procedural failure, including those requiring trans-septal puncture. Therefore, the uncrossable rate in this study may reflect a broader, more diverse patient population with varying anatomical complexities of PFOs. However, the high rate of uncrossable PFO remains a limitation of our study, and the findings should be corroborated by further studies focusing on this specific issue. As a single-center observational study with a relatively small number of patients analyzed, the results may not be generalizable to all populations. Additionally, approximately 10% of patients in the study were not evaluated using cardiac computed tomography to rule out pulmonary arteriovenous fistulas or anomalous pulmonary venous return, which may have affected the results. An additional limitation of the study is the lack of detailed investigation of the prevalence of liver disease in the patients, which is important because a right to left shunt identified on TEE in patients with undiagnosed hepatopulmonary syndrome may be mistaken for a shunt due to PFO. However, because we judged a right to left shunt with PFO only if the agitated saline contrast was detected in the left atrium within 3 cardiac cycles, we expect that the proportion of patients with hepatopulmonary syndrome in whom microbubbles are typically observed after 4 cardiac cycles is not as high as we fear. Additionally, we note a significant disparity in the duration of post-procedure follow-up between the two groups. While we strived for extended follow-up periods due to the limited number of clinical events, we acknowledge that this difference in follow-up duration poses a limitation in interpreting the study results. Future studies with larger sample sizes and more rigorous evaluation methods are needed to further investigate the long-term clinical outcomes of patients with uncrossable PFOs and to better understand the factors that contribute to recurrent stroke/TIA events in this population. Also, despite our efforts to thoroughly rule out other causes of CS, we acknowledge the possibility of excessive measures of PFO shunt grade or other biases, since this was a single center study.

## 5. Conclusions

Our study suggests that the clinical outcomes of patients with uncrossable PFOs are comparable to those of patients who undergo successful PFO closure, regardless of the baseline PFO grade. This suggests that medical therapy alone might be sufficient for preventing recurrent stroke or TIA in patients with uncrossable PFOs, even when a high-grade PFO is present. These findings may have implications for the management of patients with uncrossable PFOs, potentially reducing the need for invasive closure procedures in this population. Further research is needed to validate these findings and to optimize treatment strategies for patients with uncrossable PFOs.

## Data Availability

The datasets used and/or analyzed during the current study are available from the corresponding author on reasonable request.
