# Supplementary material for: Long-term clinical outcomes for patients with uncrossable patent foramen ovale
Source: Front Cardiovasc Med. 2023 Oct 13;10:1249259. doi: 10.3389/fcvm.2023.1249259 (PMC10611517; doi:10.3389/fcvm.2023.1249259)

**Supplemental Appendix**

**Supplemental Table 1.** Rate of Guidewire or Catheter Passage Success

**Supplemental Figure 1.** Subgroup analysis by PFO grade

Kaplan-Meier estimating the cumulative incidence of recurrent ischemic stroke or TIA in a time-to-first-event analysis.

CI, confidence intervals; HR, hazard ratio; PFO, patent foramen ovale; TIA, transient ischemic attack.

**Supplemental Table 1.** Rate of Guidewire or Catheter Passage Success

|  | **Total, % (n)** | **Grade 2, % (n)** | **Grade 3, % (n)** |
| --- | --- | --- | --- |
| Success Rate | 66.5% (163/245) | 53.4% (62/116) | 78.3% (101/129) |

**Supplemental Figure 1.** Subgroup analysis by PFO grade


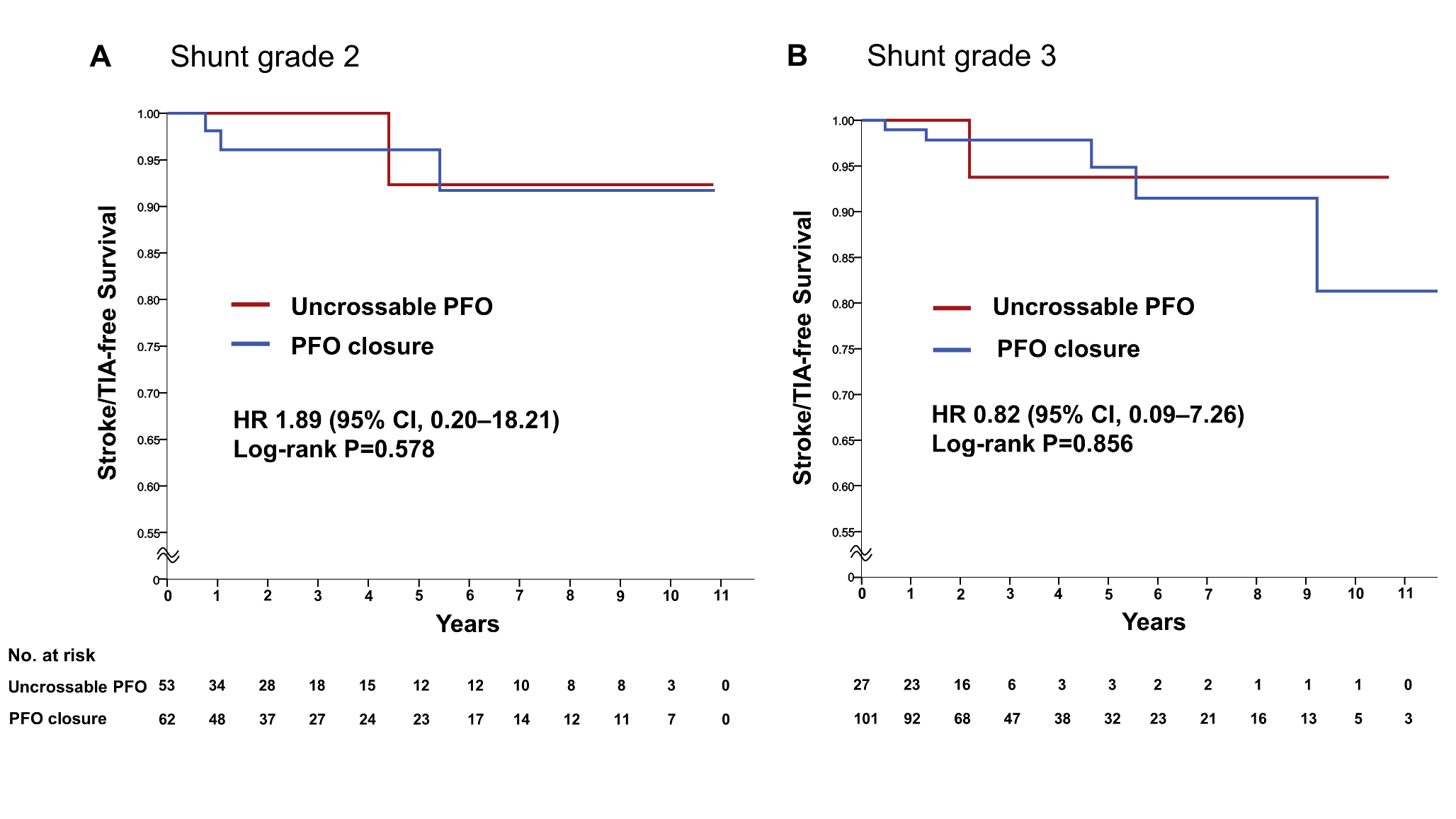

Supplement: Supplementary file 1 [file Datasheet1.docx]
