# Supplementary figures and images for: Long-term clinical outcomes for patients with uncrossable patent foramen ovale
Source: Front Cardiovasc Med. 2023 Oct 13;10:1249259. doi: 10.3389/fcvm.2023.1249259 (PMC10611517; doi:10.3389/fcvm.2023.1249259)

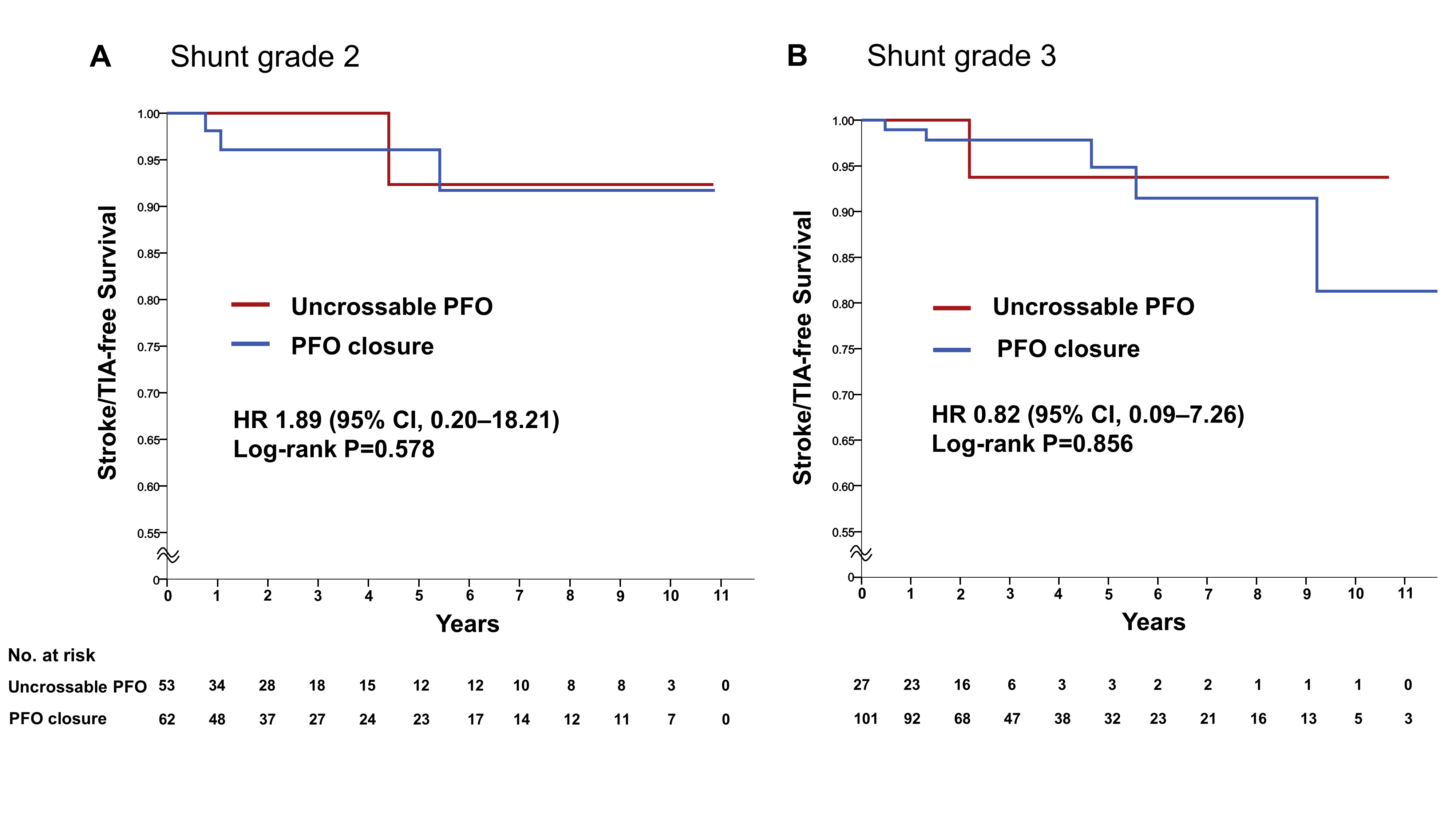

Supplement: Supplementary file 2 [file Image1.tif]
